# P-674. The Economic Burden of Infant RSV Among US Caregivers

**DOI:** 10.1093/ofid/ofae631.870

**Published:** 2025-01-29

**Authors:** Martine C Maculaitis, Cassandra Rene, Joseph C Cappelleri, Lewis Kopenhafer, Sarah J Pugh, Ronika Alexander-Parrish, Jennifer Deese, Michelle Vichnin, Monica F Turiga, Nemin Chen, Jessica E Atwell, Amy W Law

**Affiliations:** Cerner Enviza, North Kansas City, Missouri; Oracle, Jacksonville, Florida; Pfizer Inc., Groton, Connecticut; Oracle America, Inc., Los Angeles, Virginia; Pfizer, Inc., Collegeville, Pennsylvania; Pfizer, Inc, Bowie, MD; Pfizer, Cary, North Carolina; Pfizer Inc., Groton, Connecticut; Pfizer Inc., Groton, Connecticut; Oracle, Jacksonville, Florida; Pfizer, Cary, North Carolina; Pfizer, Inc., Collegeville, Pennsylvania

## Abstract

**Background:**

Respiratory syncytial virus (RSV) is the most common cause of lower respiratory infections in infants and an important cause of infant hospitalization. The burden of RSV extends to infants’ caregivers (CGs) through increased expenses, reduced earnings, and work productivity loss. This study aimed to elucidate the economic impact of providing care for an infant with RSV on CGs.

**Figure 1.**
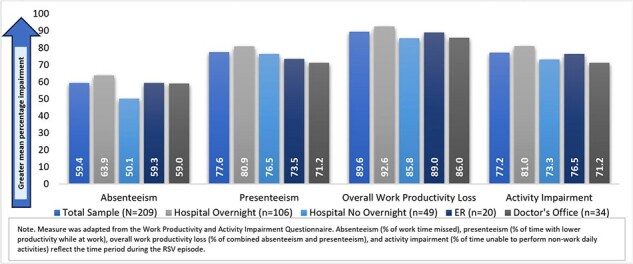
Work Productivity Loss and Non-Work Activity Impairment

**Methods:**

A cross-sectional survey of primary CGs (aged ≥18 years, US residents) of infants aged ≤12 months who had a medically attended visit for RSV in the prior 6 months between October 2023-April 2024 was conducted. CGs self-reported on sociodemographic characteristics, work productivity loss, lost workdays, healthcare resource utilization (HCRU), income loss, and total household out-of-pocket (OOP) costs based on a 6-month recall during the infant’s RSV episode. Data collection commenced in March 2024 and is ongoing. An interim analysis restricted to the subset of currently employed CGs was performed, with results summarized via descriptive statistics for the total sample and by highest level of care setting that infants received during the RSV episode (Hospital Overnight [HO], Hospital No Overnight [HNO], Emergency Room [ER], Doctor’s Office [DO]).

**Figure 2.**
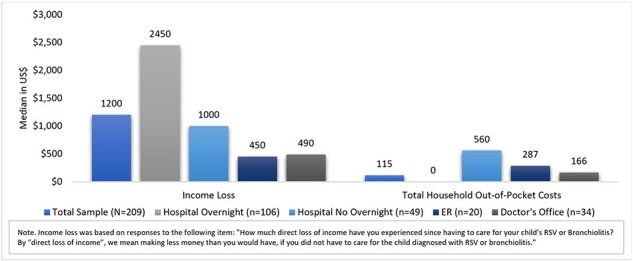
Income Loss and Total Household Out-of-Pocket Costs

**Results:**

Employed CGs (N=209) were mostly white (91%), male (57%) and had a mean age of 34 (interquartile range [IQR]: 29, 36). Most CGs reported the RSV care setting as HO (51%), followed by HNO (23%), DO (16%) and ER (10%). CGs reported a median of 2 doctor visits, 1 ER visit, and 1 hospitalization during the RSV episode. A majority (57%) reduced working hours during the RSV episode; of these, 37% stopped working completely. CGs reported a mean of 8 (IQR: 3, 8) lost workdays, with the most lost workdays reported for HO (mean: 9 [IQR: 4,8]) and ER (mean: 8 [IQR: 4, 10]) care settings. Mean absenteeism, presenteeism, overall work productivity loss, and activity impairment, as well as median income loss, were highest for HO care setting; CGs incurred a median of $115 in total household OOP costs and $1200 in income loss (**Figures 1**-**2**).

**Conclusion:**

Economic burden among CGs was substantial across care settings, with the highest burden among HO. Protecting infants from severe RSV illness can also reduce the economic burden for CGs.

**Disclosures:**

**Martine C. Maculaitis, PhD**, Oracle Life Sciences: Employee of Oracle Life Sciences, which received funding from Pfizer to conduct the study. **Cassandra Rene, PhD, MPH**, Pfizer Inc.: Advisor/Consultant **Joseph C. Cappelleri, PhD**, Pfizer Inc.: Employee|Pfizer Inc.: Stocks/Bonds (Public Company) **Sarah J. Pugh, PhD, MPH**, Pfizer Inc.: Employee|Pfizer Inc.: Stocks/Bonds (Public Company) **Ronika Alexander-Parrish, RN, MAEd**, Pfizer, Inc.: Employee|Pfizer, Inc.: Ownership Interest|Pfizer, Inc.: Stocks/Bonds (Private Company) **Jennifer Deese, PhD, MPH**, Pfizer: Stocks/Bonds (Private Company) **Michelle Vichnin, MD**, Pfizer Inc.: Employee|Pfizer Inc.: Stocks/Bonds (Public Company) **Monica F. Turiga, MD**, Pfizer Inc.: Employee|Pfizer Inc.: Stocks/Bonds (Public Company) **Nemin Chen, PhD**, Pfizer: Advisor/Consultant **Jessica E. Atwell, PhD, MPH**, Pfizer Inc.: Employee|Pfizer Inc.: Stocks/Bonds (Public Company) **Amy W. Law, PharmD**, Pfizer Inc.: Employee|Pfizer Inc.: Stocks/Bonds (Public Company)

